# Spontaneous Posterior Subdural Pyogenic Escherichia coli Abscess Secondary to Lumbar Spondylodiscitis

**DOI:** 10.7759/cureus.13703

**Published:** 2021-03-04

**Authors:** Joseph J Gleeson, Andrew J Berg, Peter R Loughenbury, Senthil K Selvanathan, Andraay Leung

**Affiliations:** 1 Spinal Surgery, Leeds Teaching Hospitals NHS Trust, Leeds, GBR

**Keywords:** spinal subdural abscess, e. coli, spinal abscess surgery, spondylodiscitis, spinal mri, laminectomy, spinal decompression, gram negative bacteremia

## Abstract

Pyogenic subdural spinal collections are rare but an important pathology to recognise and manage appropriately. We report the case of a 56-year-old female who developed a posterior subdural spinal collection associated with local discitis. There was no direct communication between the infected disc and subdural space, and the collection was located posteriorly within the subdural space which makes this case all the more unusual.

We discuss the need for spinal subdural collections to be considered as a differential in patients with back pain and lower limb neurology (especially when there is a known spinal infective focus), the importance of careful interpretation of imaging, and the pathophysiological mechanisms and organisms known to cause spinal subdural collections.

## Introduction

Subdural spinal collections are intradural extramedullary pyogenic infections in the spine. They are exceptionally rare, with only around 100 cases reported to date [1]. Causes of spinal subdural collections previously reported have included bacteraemia with haematogenous spread, direct extension from a local discitis, and iatrogenic spread [1]. The organism most commonly responsible is *Staphylococcus aureus* [2], although other organisms have been reported including *Escherichia coli* and *Streptococci *species [3].

We report the only case, to our knowledge, of a patient developing a spontaneous posterior subdural, extramedullary spinal collection in association with *E. coli* discitis, but without direct communication between the infected disc and the subdural space.

## Case presentation

A 56-year-old female was admitted with a one-week history of uncontrollable lower back pain radiating to her buttocks and thighs, and a one-day history of fever. Her past medical history included hypertension and alcohol excess. There was no neurological deficit on examination. Laboratory investigations revealed elevated white cell count and C-reactive protein, and deranged liver function tests.

The patient was admitted under the infectious diseases team, and treatment was commenced for presumed cholecystitis on accounts of the abnormal liver function tests, with cefuroxime and metronidazole. Other differential diagnoses including pyelonephritis and discitis were considered. Abdominal ultrasound, magnetic resonance cholangiopancreatography (MRCP), and urine culture, however, showed no evidence of infective pathology. Blood cultures were positive for *E. coli*, sensitive to amoxicillin. Antibiotics were therefore converted to high dose intravenous amoxicillin.

**Figure 1:**
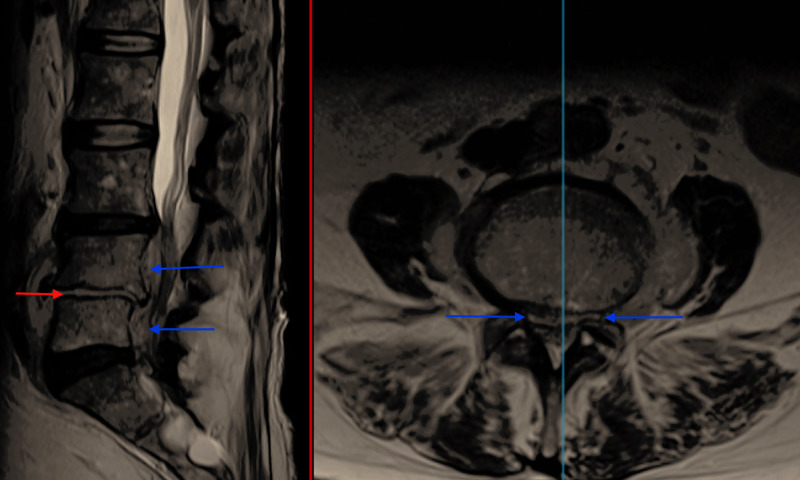
T2 TSE Sagittal and Axial MRI showing fluid in the L4/5 disc space (red arrow) and vertebral body oedema consistent with discitis and osteomyelitis along with an anterior epidural collection (blue arrows). TSE - Turbo Spin Echo; MRI - Magnetic Resonance Imaging

Magnetic resonance imaging (MRI) of the spine was obtained and identified an L4/L5 spondylodiscitis with osteomyelitis (Figure 1).

A fluid collection tracking anteriorly in the epidural space at L4 and L5 was also observed. While this collection caused severe central canal stenosis and compression of the cauda equina, there were no clinical signs of cauda equina syndrome.

Despite blood culture-guided antibiotics, the patient continued to be pyrexial, repeat blood cultures were persistently positive for *E. coli*, and inflammatory markers remained raised. Symptomatically she deteriorated with worsening back pain and new right hip pain. Further MRI of the spine and hip revealed progression of discitis and an anterior soft tissue collection extending into the psoas muscles bilaterally. CT-guided aspiration of the psoas collection obtained further growth of *E. coli*.

**Figure 2:**
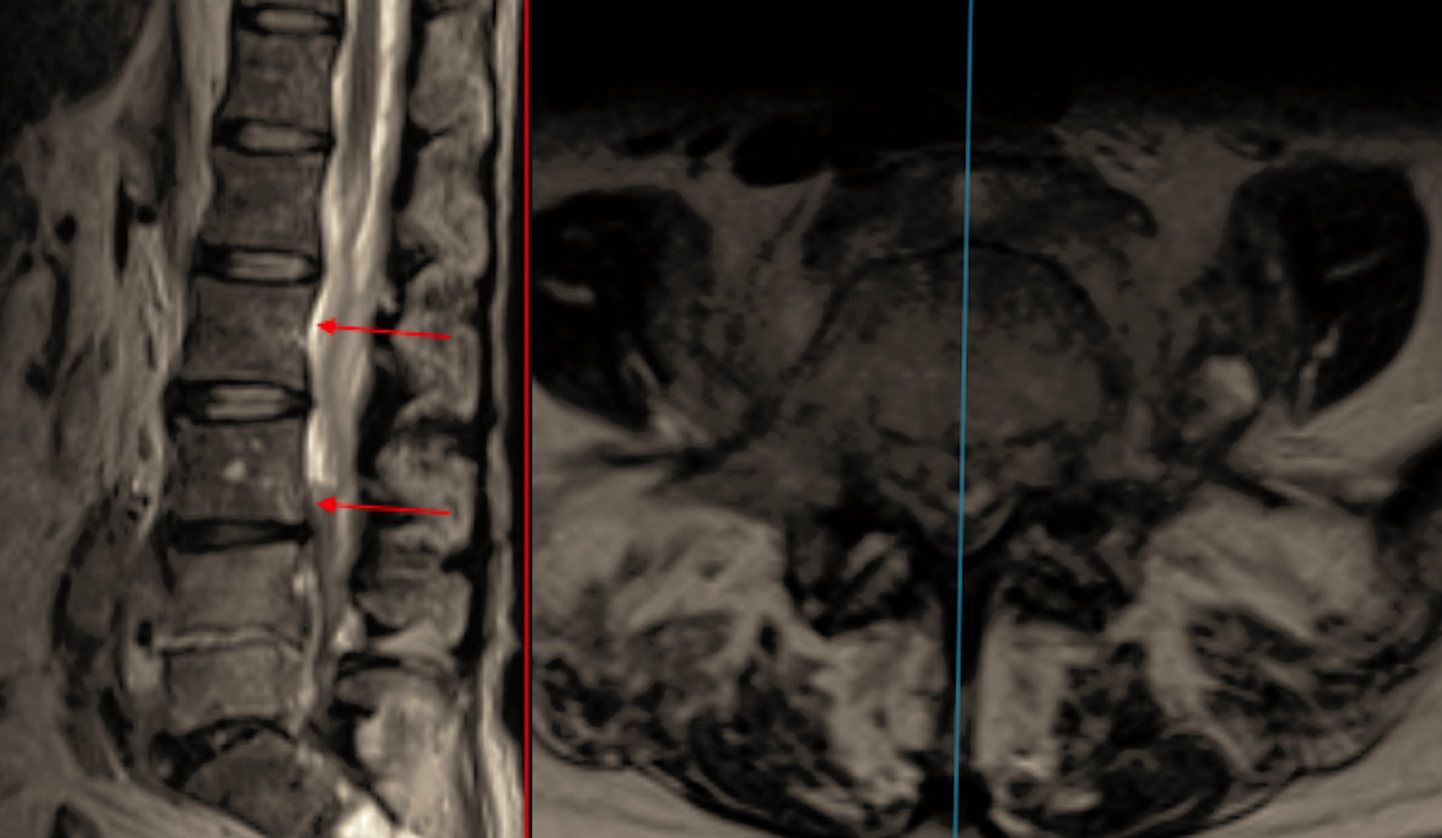
T2 TSE Sagittal and Axial MRI showing worsening of the epidural collection with proximal extension (red arrows). TSE - Turbo Spin Echo; MRI - Magnetic Resonance Imaging

Given the progression, antibiotics were changed to cefotaxime. Despite the alteration in medical management her symptoms continued to deteriorate, and further MRI was obtained revealing further progression of the infection with a large epidural collection extending up to T11, and marked canal stenosis between L2/3 and L5/1 (Figure 2).

Given the failure of medical management, she was transferred to the care of the spinal surgery department where she underwent L4/5 instrumented fusion and decompression. Intra-operatively, sloughy tissue was encountered around the flavum and dura, but there was no frank abscess and no breach of the dura.

On the first post-operative day, the pyrexia and the leg and back pain all improved. However, on the second post-operative day she experienced sudden onset bilateral radicular pain in an L3 dermatomal pattern and her pyrexia recurred. Lower limb neurological assessment remained unremarkable.

**Figure 3:**
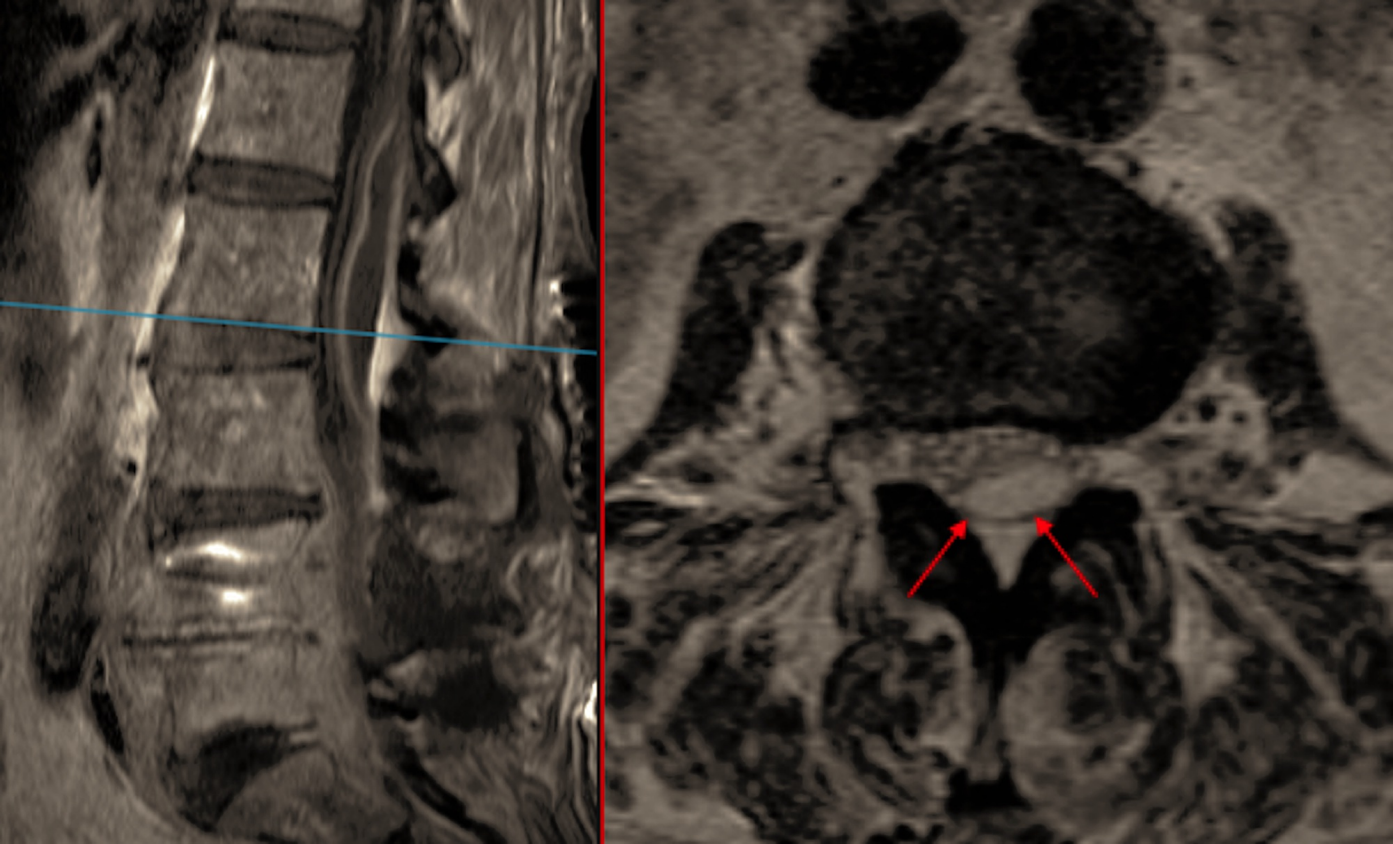
T1 TSE with Gadolinium and Axial T2 TSE show subdural collection posteriorly at L1 – L3 levels. Normal epidural fat is visible posteriorly with plane indicating dura (red arrow). TSE - Turbo Spin Echo

An urgent MRI whole spine was conducted, showing abnormal signal posteriorly within the dural sac from L1-3. Close examination of these images indicated that the epidural fat plane remained intact and that the collection was subdural. Differential diagnosis at this stage included subdural hemorrhage, subdural infection, and arachnoiditis. Additional post-contrast MRI sequences demonstrated a subdural collection extending from L3 proximally, exerting most pressure at L1 and L2 with compression of the cauda equina (Figure 3).

The patient returned to theatre for an L2 laminectomy and drainage of the subdural collection. Durotomy was performed, leaving the arachnoid intact, and pus was expressed under pressure. After washout, the dura was left open to avoid re-accumulation of the collection. Haemopatch (Baxter Healthcare Corporation, Deerfield, IL, USA) was used to seal the durotomy.

**Figure 4:**
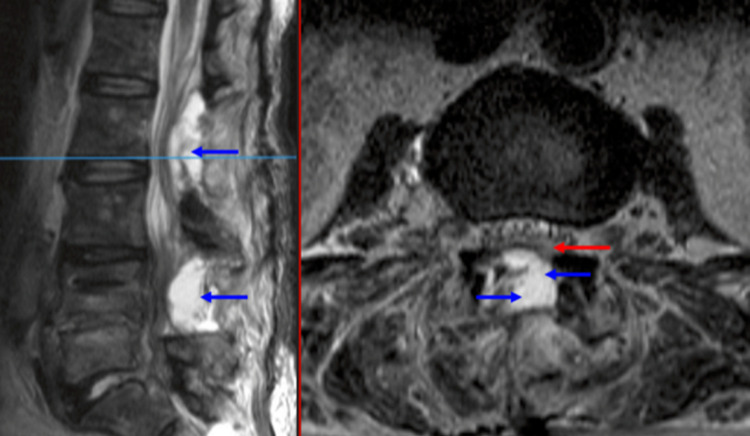
Post-operative T2 TSE Sagittal and Axial MRI showing posterior fluid collections in keeping with recent surgery (blue arrows) and reduction in subdural collection (red arrows). TSE - Turbo Spin Echo; MRI - Magnetic Resonance Imaging

Following the second operation, she remained neurologically intact and her leg pain improved. Repeat MRI imaging showed reduction of the subdural collection (Figure 4). After two weeks of intravenous antibiotics, she was converted to oral ciprofloxacin, and completed six weeks of antibiotics from the date of the second surgical procedure.

## Discussion

Subdural pyogenic collections are extremely rare but should be considered as a differential diagnosis in patients presenting with back pain and neurological deterioration in the lower limbs. Index of suspicion should be raised when there is a known infective focus in the spine and a failure to respond to medical management.

This case demonstrates that a worsening of symptoms, failure of clinical improvement, or deterioration in neurological symptoms or signs should prompt further investigation with whole spine three-dimensional imaging. Images obtained must be carefully interrogated and in particular, the location of collections in relation to the normal epidural fat plane should be appreciated to help differential intra-dural from extra-dural collections.

The majority of reported cases of subdural spinal collections have been in the lumbar spine. Radicular pain in the lower limbs and other neurological sequelae of lumbar nerve root compression are described. Thoracic subdural collections have also been reported [3,4]. Previous reports have described a number of pathophysiological mechanisms including haematogenous spread from distant collections [1], iatrogenic causes (following spinal injections [5] or inadvertent durotomies during spinal surgery [6]), direct extension of deep spinal infection [1], direct spread via a pressure ulcer [7] and direct extension from a spondylodiscitis via an epidural abscess [8].

In the case presented here, there was no direct extension of infection and the subdural collection occurred without direct communication to the pyogenic disc space infection or epidural extension. We postulate that, while the patient did have discitis, it appears the causative pathogen was transmitted to the subdural space via haematogenous spread rather than direct extension.

A variety of causative pathogens have been described as causing subdural collections. Ramos et al. in their review of 66 reported cases of subdural infections report *Staphylococcus aureus *was identified in 56.1%, anaerobes in 6%, *E. coli*, beta-hemolytic* Streptococci*, *Streptococcus viridans*, *Streptococcus milleri, *and *Mycobacterium tuberculosis *in 3%, and a variety of other organisms in individual cases. In 13.6% of cases the organism was not identified [1].

In our case the causative organism was *E. coli*, with several blood cultures positive for *E. coli*, and growth in the samples from the psoas collection. *E. coli* is a less common cause of spinal subdural collections although has been noted in previous case reports [8].

## Conclusions

In conclusion, subdural spinal collections are a rare but important pathology. *E. coli* is an unusual but possible causative agent. Careful interpretation of MRI imaging is the key to diagnosing this pathology, and prompt surgical intervention is required.
